# Protective role of Dihydromyricetin in Alzheimer’s disease rat model associated with activating AMPK/SIRT1 signaling pathway

**DOI:** 10.1042/BSR20180902

**Published:** 2019-01-03

**Authors:** Ping Sun, Jun-Bo Yin, Li-Hua Liu, Jian Guo, Sheng-Hai Wang, Chun-Hui Qu, Chun-Xia Wang

**Affiliations:** Qingdao Mental Health Center, 229 Nanjing Road, Qingdao 266034, Shandong, P.R. China

**Keywords:** AMPK, Alzheimer’s disease, cognitive function, Dihydromyricetin, inflammatory responses, SIRT1

## Abstract

The aim of the present study was to understand the possible role of the Dihydromyricetin (DHM) in Alzheimer’s disease (AD) rat model through regulation of the AMPK/SIRT1 signaling pathway. Rats were divided into Sham group, AD group, AD + DHM (100 mg/kg) group and AD + DHM (200 mg/kg) group. The spatial learning and memory abilities of rats were assessed by Morris Water Maze. Then, the inflammatory cytokines expressions were determined by radioimmunoassay while expressions of AMPK/SIRT1 pathway-related proteins by Western blot; and the apoptosis of hippocampal cells was detected by TdT-mediated dUTP nick end labeling assay. AD rats had an extended escape latency with decreases in the number of platform crossings, the target quadrant residence time, as well as swimming speed, and the inflammatory cytokines in serum and hippocampus were significantly elevated but AMPK/SIRT1 pathway-related proteins were reduced. Meanwhile, the apoptosis of hippocampal cells was significantly up-regulated with decreased Bcl-2 and increased Bax, as compared with Sham rats (all *P*<0.05). After AD rats treated with 100 or 200 mg/kg of DHM, the above effects were significantly reversed, resulting in a completely opposite tendency, and especially with 200 mg/kg DHM treatment, the improvement of AD rats was more obvious. DHM exerts protective role in AD via up-regulation of AMPK/SIRT1 pathway to inhibit inflammatory responses and hippocampal cell apoptosis and ameliorate cognitive function.

## Introduction

As a major type of dementia in aging population, Alzheimer’s disease (AD) has been widely deemed as a multifactorial degenerative disease in central nerve system with the characteristics of progressive cognitive decline or dysfunction [1,2]. At present, AD, only secondary to the cardiovascular disease or tumors, has risen to the third place among all diseases, costly and severely distressing for patients and their families [3–5]. AD, as the increasing epidemic threatening public health, is quite complicated mainly involving two neuropathological hallmarks: senile plaques and neurofibrillary tangles, which consisted of amyloid-β (Aβ) peptides and hyperphosphorylated tau protein, respectively [6,7]. As reported, the deposition and aggregation of the Aβ peptide have been shown on numerous occasions, which could trigger inflammatory responses and cause neuronal dysfunction, and ultimately resulting in dementia or AD [8,9]. Although much progress has been made in the AD treatment in recent years, the efficacy is not very satisfactory. Thus, understanding the molecular mechanism of AD is of great significance for AD treatment [10].

Dihydromyricetin (DHM), also known as ampelopsin (AMP), is a kind of natural flavonoids compounds and the major functional ingredient of *Ampelopsis grossedentata*, with physiological functions like anti-inflammation, anti-oxidation and enhancement in immunity [11]. As demonstrated previously, DHM can activate the AMPK signaling pathway to induce the autophagy, and thereby improving skeletal muscle insulin resistance and hyperglycemia-induced cell injuries [12,13]. Moreover, Kou et al. [14] pointed out that DHM can also alleviate the skeletal muscle atrophy in aging rats through activation of AMPK/SIRT1/PGC-1α signaling pathway. As for AMP-activated protein kinase (AMPK), it is a member of serine/threonine protein kinase extensively existing in eukaryotic cells, which could be acted as an important energy sensor to maintain the cellular energy balance [15]. Recently, AMPK was suggested to play a role in controlling the aging process [16]. In terms of SIRT1, a NAD^+^-dependent histone deacetylase, it is a major downstream molecule in AMPK signaling pathway, with a close correlation to the pathogenesis of aging-related diseases, like neurodegenerative diseases [17]. Of note, Shah et al. [18] revealed the involvement of AMPK/SIRT1 signaling pathway in the modulation of Aβ deposition and cognitive functions in AD rats. As such, we speculated that the potential regulatory role of DHM in the AMPK/SIRT1 signaling pathway might be crucial to the aging-related diseases, like AD.

Therefore, using the AD model of rats, the present study was done to verify whether DHM can exert the regulatory roles in AD through mediating the AMPK/SIRT1 signaling pathway, in order to provide potential strategy for the clinical treatment of AD.

## Materials and methods

### Ethics statement

All animal work described in the present study was approved by the Ethic Committee of Qingdao Mental Health Center. The protocols regarding the animal experiments were stipulated and implemented in strictly accordance with the Guide for the Care and Use of Laboratory Animals stipulated by National Institute of Health (NIH) [19].

### Model construction and grouping

A total of 40 pathogen-free Sprague Dawley (SD) male rats (age: 8 weeks; bodyweight: 250–300 g) were obtained from Shanghai SLAC Laboratory Animal Co. Ltd. and raised in the laboratory for 1 week of adaption in a 12/12 light/dark cycle with the temperature of 21 ± 2°C and free access to water and food. Then, rats were randomly divided into Sham group, AD group, AD + DHM (100 mg/kg) group and AD + DHM (200 mg/kg) group, with 10 rats per each group. AD models were established as follows: rats were anesthetized of 10% chloral hydrate (300 mg/kg) by intraperitoneal injection and fixed in the stereotaxic apparatus in prone position. Then, the intrahippocampal injection of amyloid-β_1–42_ (Aβ_1–42_) oligomers (5 μl, Sigma, St. Louis, MO, U.S.A.) was performed bilaterally using the micro-syringe with coordinates (from bregma): anteroposterior, 3 mm; mediolateral, 2 mm; dorsoventral, 3.5 mm [20]. The validity of AD model was indicated by severe cognitive deficit, impaired hippocampal synaptic plasticity and marked increase levels of β-site amyloid precursor protein (APP) cleaving enzyme [21], and all rat injected with Aβ_1–42_ were confirmed to be successfully established of the model. While 5 μl of 0.9% normal saline instead of Aβ solution was injected in rats from the Sham group. Two hours prior to the Aβ infusions, rats in the AD + DHM (100 mg/kg) group and AD + DHM (200 mg/kg) group were additionally received the intraperitoneal injection of DHM in doses of 100 and 200 mg/kg, respectively. The dosage of the DHM was chosen according to a previous study [14]. Drug administration lasted for 21 days. DHM (CAS No. 27200-12-0) purified by high-performance liquid chromatography with the purity of more than 98% was purchased from Zelang Medical Technological Co. Ltd. (Nanjing, China). Chemical structure and chromatograms of DHM were shown in Supplementary Figure S1.

### Morris water maze

Place navigation test was performed immediately after the drug administration. The MWM task lasted for 5 days. The first day represented the place navigation test. In brief, rats were placed in the water with their head facing the wall, randomly from any of four quadrants, and the time(s) of rats searching for the platform was recorded. Any rats failed in arriving at the platform in 120 s were guided to the platform, allowing them to stay on the platform for 30 s, and their stay in water was recorded as 120 s. After test, rats were dried before being placed into the cages. After consecutive 5 days of training (twice per day), we recorded the time(s) of rats in searching for the platform in each quadrant, with the average of escape latencies in four quadrants as the final latency of this day. Spatial probe test was carried out at the sixth day with no platform in the maze, and rats were placed in a labeled place in the quadrant. In this test, we recorded the times that rats passed through the position of platform. This test was performed twice a day (morning and afternoon), and the average was used as the final result, and the swimming speed was recorded.

### Sample preparation

After MWM, five rats in each group were anesthetized by intraperitoneal injection of chloral hydrate. Through the incision in the chest, the heart was exposed, where a small incision was made at the apex for inserting a perfusion needle. Right auricle was removed, and 150 ml of 0.9% normal saline was perfused rapidly, followed by perfusion with 300 ml of 4% neutral paraformaldehyde (PFA). Brain tissues were taken out and placed in 4% neutral PFA. The remaining five rats were decapitated under anesthesia to collect the brain tissues, from which the hippocampus was taken out rapidly on ice and preserved at –80°C for further use.

### Radioimmunoassay

Under anesthesia, blood samples were collected from the heart, and after centrifugation, supernatant was transferred in new Eppendorf (EP) tubes for further use. Meanwhile, homogenate of hippocampus was also centrifuged to obtain the supernatant. Interleukin (IL)-1β, tumor necrosis factor (TNF)-α and IL-6 levels were detected in the serum and hippocampus tissues of rats in strictly accordance with the instructions of radioimmunoassay kit.

### Hematoxylin & eosin and Congo Red staining

Brain tissues fixed in 4% neutral PFA were embedded in paraffin and sliced serially into 4-μm-thickness sections. Following dewaxing in xylene and hydrating with alcohol in gradient concentrations, sections were rinsed using tap water and stained with hematoxylin (Biohao Biotechnology Institute, Wuhan, China) for 1 min. Residual hematoxylin was rinsed using tap water. Sections were differentiated using 1% hydrochloric acid-ethanol, blued in 1% ammonia hydroxide for 30 s, stained with eosin for 2 min, dehydrated with alcohol in gradient concentrations, fixed in xylene and sealed with neutral balsam. The morphological changes were observed under a microscope. The experiment was repeated three times. For Congo Red staining, deparaffinized and rehydrated sections were first stained in Gill’s hematoxylin solution (Sigma, St. Louis) for 10 min and then rinsed in running tap water for 5 min and incubated in alkaline sodium chloride solution for 20 min. Sections were then stained in Congo Red working solution (Sigma) for 15 min, followed by dehydration through 95% alcohol. They were then dehydrated, hyalinized and mounted for microscopic examination.

### TUNEL methods

Brain sections were fixed in 4% neutral PFA and taken out for paraffin embedding. Then, sections were dewaxed in xylene and hydrated in ethanol in gradient concentrations. Following rinsing sections in tap water, antigen retrieval was implemented at 80°C. Thereafter, the sections were incubated in presence of Protease K (Sigma, U.S.A.) and then placed in TDT buffer (Sigma, U.S.A.) for pre-incubation. After washed with PBS again, the sections were then incubated with anti-digoxin and anti-serum alkaline phosphatase complex (Sigma, U.S.A.) at 37°C overnight. Following washes in Tris-buffer, counter staining was performed with the corresponding reagent, and ended using Tris-buffer. Apoptosis in hippocampus of rats was observed under a microscope, and the results were quantified in Image-Pro Plus. Apoptotic ratio is calculated using the formula: Apoptotic ratio = TUNEL-positive cell quantity/total quantity of cells. The experiment was repeated three times.

### Western blot assay

Following snap freezing in liquid nitrogen, hippocampus lysate was prepared using radioimmunoassay reagent for 30 min and centrifuged at 4°C for 30 min at 12000 ***g***. Protein concentration was measured using the bicinchoninic acid (BCA) kit (Beyotime, Beijing, China). Protein extracts, together with loading buffer, were heated at 95°C for 10 min, and according to the relative molecular weight of targeted proteins, 6–12% separation gel was prepared (Boster, Wuhan, China) for separation of proteins. Then, proteins on the gel were transferred onto the polyvinylidene fluoride (PVDF) membrane (Millipore, U.S.A.) that were then blocked with 5% bovine serum albumin for 1 h at room temperature. Membranes were then incubated with the primary antibodies, such as anti-AMPK, anti-p-AMPK, anti-SIRT1, anti-Bcl-2, anti-Bax, anti-NF-κB p65 and anti-GAPDH (Cell Signaling Technologies, U.S.A.) at 4°C overnight. After washed with Tris-buffer saline with Tween 20 for three times (5 min/time), blots were then probed with the corresponding HRP-conjugated secondary antibodies at room temperature for 1 h. The target protein was visualized by the enhanced chemiluminescence (ECL) reagent (Beyotime, Beijing, China). Targeted blots were quantified by ImageJ software with GAPDH as internal control. The experiment was repeated three times.

### Statistical methods

All data were analyzed using SPSS 21.0 software (SPSS Inc, Chicago, IL, U.S.A.). Measurement data, in form of mean ± standard deviation ($\bar{x}$ ± *s*), were compared by *t*-test between groups. One-way ANOVA test was used to compare differences among multiple groups followed by Tukey’s HSD Post Hoc test to compare significance between groups. *P*<0.05 considered as being statistically significant.

## Results

### Comparison of the spatial learning and memory abilities of rats in different groups

**Figure 1:**
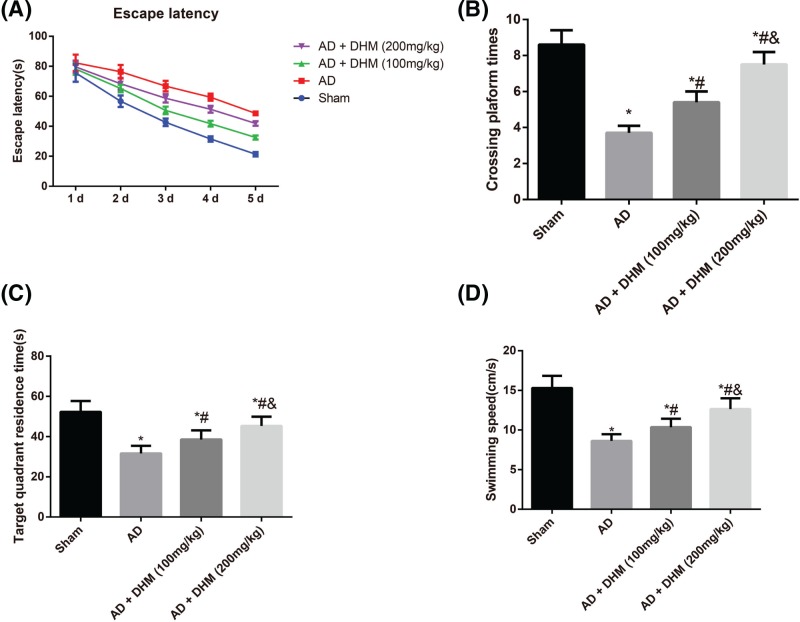
Examination of the spatial learning and memory abilities of rats by MWM Comparison of the escape latency of rats (**A**), the numbers of platform crossings (**B**), target quadrant residence time (**C**) and the swimming speed (**D**) in different groups. Data were indicated as mean ± standard deviation values (*n*=10). *P<0.05 vs*.* the Sham group; ^#^*P*<0.05 vs. the AD group; ^&^*P*<0.05 vs. the AD + DHM (100 mg/kg) group.

As shown in Figure 1, the AD rats had evident decreases in spatial exploration ability and learning and memory abilities, as evident by the prolonged escape latency, reduced numbers of platform crossings, shortened target quadrant residence time and decreased swimming speed, when compared with that in the Sham group (all *P*<0.05). However, after 100 or 200 mg/kg of DHM treatment in AD rats, the shortened escape latency, increased numbers of platform crossings, prolonged target quadrant residence time, as well as increased swimming speed, were observed (*P*<0.05), and these changes was more obvious in AD rats treated with 200 mg/kg of DHM (*P*<0.05).

### Effects of DHM on the morphological changes of rats in different groups

**Figure 2:**
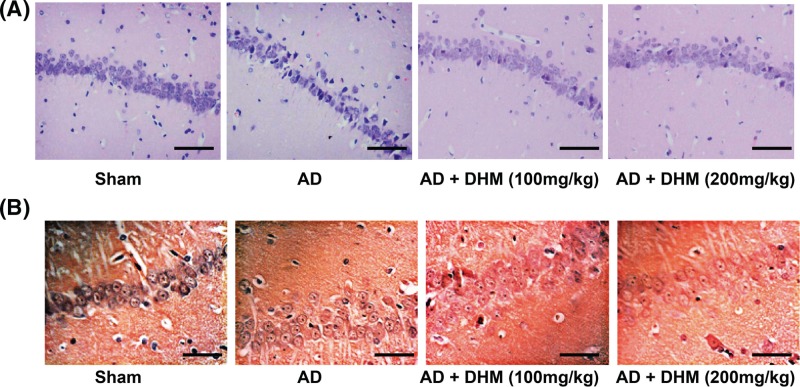
The morphological changes of the hippocampus of rats by HE(A) and congo red staining (B) (×400, *n*=5) Scale bar = 50 μm.

Morphological changes in the hippocampus of rats were observed by using hematoxylin & eosin (HE) staining, and the results are listed in Figure 2A. In the Sham group, the hippocampal neurons of rats were lightly stained for intracellular structures and nucleus. In contrast, the layers and numbers of hippocampal cells of the AD rats were decreased with enlarged intercellular space and disordered cells; especially, some cells exhibited shrink in volume, with pyknosis or rupture in nuclei, and cells were deeply stained into red. After AD rats treated with 100–200 mg/kg DHM, the hippocampal neurons were evenly stained and arranged closely, and the neuron cells were increased, which was more significant amelioration in those treated with 200 mg/kg of DHM. Congo Red staining (Figure 2B) was used to determine the amyloid plaques deposition in the hippocampus. Amyloid plaques exhibited a light red dispersion without distinct boundaries and the amount of amyloid deposition in the AD rats appeared to be increased as compared with the hippocampi of the Sham group. Following DHM treatment at two different doses, the cells were stained lighter with clear cellular structure as compared with the rats in the AD group.

### Comparison of the inflammatory cytokines in serum and hippocampus of rats in different groups

**Figure 3:**
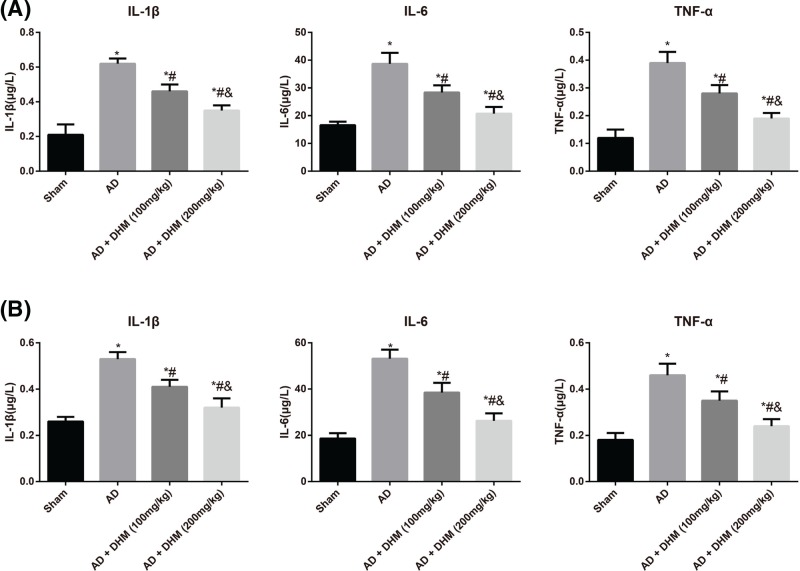
Comparison of the inflammatory cytokines in serum and hippocampus of rats in different groups Data were indicated as mean ± standard deviation values (*n*=5); *P<0.05 vs. the Sham group; ^#^*P*<0.05 vs. the AD group; ^&^*P*<0.05 vs. the AD + DHM (100 mg/kg) group.

Compared with the Sham group, rats in the AD group had significantly increased in the levels of IL-1β, IL-6 and TNF-α in serum and hippocampus (all *P*<0.05, Figure 3). Nevertheless, AD rats treatment with DHM (100 mg/kg) group and DHM (200 mg/kg) significantly reduced the serum and hippocampus levels of IL-1β, IL-6 and TNF-α in rats, and the reductions in the AD + DHM (200 mg/kg) group were much more evident than those in the former group (all *P*<0.05).

### Comparison of the hippocampal neuronal apoptosis of rats in different groups

**Figure 4:**
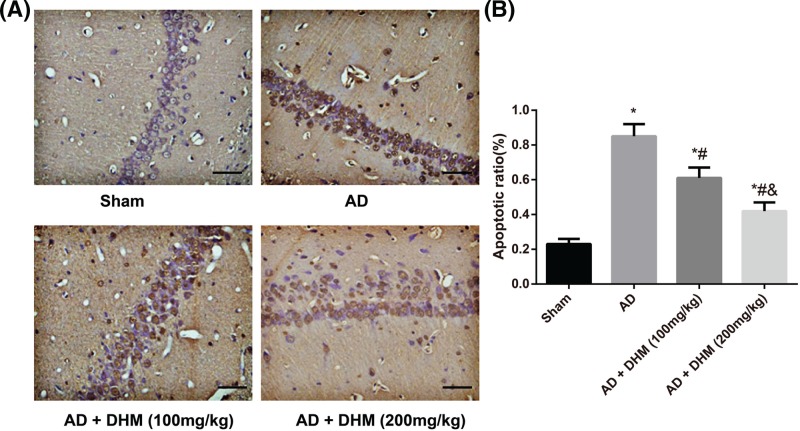
Detection of the hippocampal neuronal apoptosis of rats in different groups by TUNEL staining (×400); scale bar = 50 μm (**A**) Detection of the hippocampal neuronal apoptosis of rats in different groups using TUNEL staining. (**B**) Comparison of the apoptotic ratio of rats in different groups. Data were indicated as mean ± standard deviation values (*n*=5); *P<0.05 vs*.* the Sham group; ^#^*P*<0.05 vs. the AD group; ^&^*P*<0.05 vs. the AD + DHM (100 mg/kg) group.

TdT-mediated dUTP nick end labeling (TUNEL) staining method was applied to determinate the hippocampal neuronal apoptosis of rats in different groups (Figure 4). In comparison with the Sham group, rats in the AD group had a significant increase in the apoptotic ratio of the hippocampus (*P*<0.05). When compared with the AD group, the apoptotic ratios of the hippocampus in rats from the AD + DHM (100 mg/kg) group and AD + DHM (200 mg/kg) group were significantly decreased, and especially lower in AD rats with 200 mg/kg of DHM treatment (all *P*<0.05).

### Expressions of AMPK/SIRT1 signaling pathway-related protein in rats

**Figure 5:**
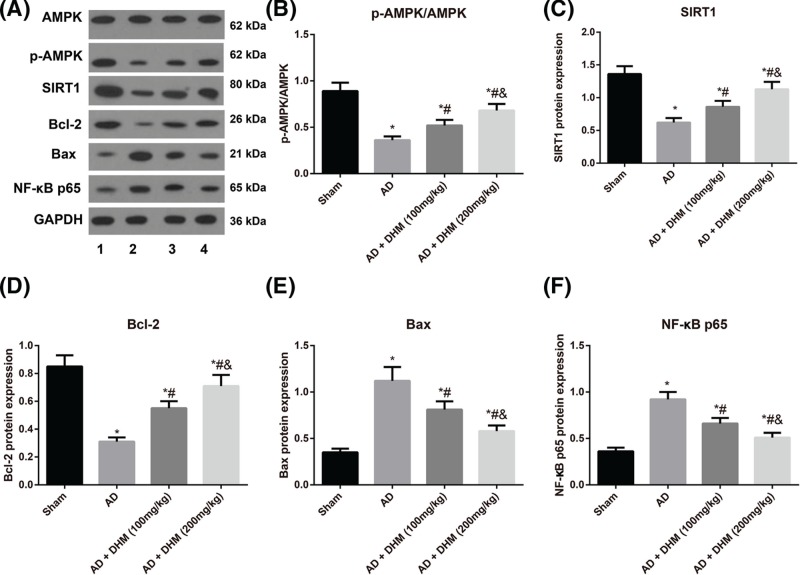
Expressions of AMPK/SIRT1 signaling pathway-related protein in rats detected by Western blot (**A**) Expressions of AMPK/SIRT1 signaling pathway-related protein in rats detected by Western blot, 1, Sham group, 2, AD group, 3, AD + DHM (100 mg/kg) group, 4, AD + DHM (200 mg/kg) group. (**B**–**F**) Comparisons of the protein expressions of p-AMPK/AMPK, SIRT1, Bcl-2, Bax and NF-κB p65 of rats in different groups. *P<0.05 vs. the Sham group; ^#^*P*<0.05 vs. the AD group; ^&^*P*<0.05 vs. the AD + DHM (100 mg/kg) group.

As shown in Figure 5, the protein expressions of p-AMPK/AMPK and SIRT1 in the hippocampus of rats from the AD group exhibited a significant decline, with an evident reduction of Bcl-2 and elevations of Bax and NF-κB p65 (all *P*<0.05). These changes were rescued after administration of either 100 or 200 mg/kg of DHM, which had more evident alterations in rats with 200 mg/kg of DHM.

## Discussion

Intracerebroventricular injection of Aβ_1–42_ has been shown to induce the behavioral and pathological characteristics of AD, like learning deficits and hippocampal damage, which has been used as a common method for the preparation of AD models [22,23]. Aβ_1–42_, developed from the partial hydrolysis of APP, has been thought to be a critical step in AD pathogenesis, since its accumulation led to amyloid fibril formation and the generation of senile plaques [24,25]. Therefore, in the present study, the AD rat models induced by Aβ_1–42_ in our study had the prolonged escape latency with decreases in the number of platform crossings and the percentage time spent in the target quadrant, which was consistent with the previous observations [26,27], exhibiting an evident cognitive impairment in AD rats. Meanwhile, HE staining also revealed the pathological alterations of hippocampal cells, which confirmed the successful establishment of AD models through the behavioral and pathological perspectives. However, after treated with DHM, either 100 or 200 mg/kg (these concentrations have been confirmed to be safety), the learning and memory abilities of AD rats were elevated with significant alleviations in the morphological and pathological changes of hippocampus, suggesting that DHM may protect rats from the Aβ_1–42_-induced cognitive dysfunction. Furthermore, the serum and hippocampus levels of inflammatory cytokines in rats were then determined by radioimmunoassay, and consequently, the significant increased levels of IL-1β, IL-6 and TNF-α were observed in the serum and hippocampus of AD rats. Worth mentioning, AD-related inflammatory reaction was partially associated with the activation of microglial cells and astrocytes surrounding the Aβ deposits [28,29]. Moreover, microglial cells, a kind of immunocytes, could secrete the inflammatory cytokines, including IL-6 and TNF-α, when activated; while the activation of astrocytes also triggered the massive generation of inflammatory mediators, like IL-1β, IL-6 and TNF-α etc. [30]. And these inflammatory cytokines usually resulted in neuronal necrosis and lesions, giving rise to cognitive dysfunction [31]. Importantly, the serum and hippocampus levels of inflammatory cytokines were obviously decreased in rats with DHM treatment. Similarly, DHM can also alleviate the LPS-induced neuroinflammation in microglial cells through suppression of nuclear factor-κB (NF-κB) and JAK2/STAT3 pathway, as indicated by Weng et al. [32], showing that DHM may have an anti-inflammatory effect on Aβ_1–42_-induced neuroinflammatory responses.

In addition, inhibition of AMPK/SIRT1 signaling pathway was further discovered in AD rats but was reversed after different doses of DHM treatments. In the study of Kou et al., DHM can also activate the AMPK/SIRT1/PGC-1α signaling pathway in D-gal-induced aging rats [14]. There was evidence believed that the modulation effect of AMPK/SIRT1 pathway was mainly achieved by the down-regulation of transcript factors (NF-κB and AP-1), as well as the acetylation of histone [33]. As we know, the increased acetylation of the NF-κB p65 subunit has been linked to the activation of NF-κB, which was a crucial transcript factor in inflammatory responses, and NF-κB activation can trigger the expression of pro-inflammatory cytokines, like TNF-α [34]. Recently, SIRT1 has been suggested to directly act on NF-κB, and to decrease the acetylation of p65 (Lys310), thereby suppressing the transcript activity and pro-inflammatory expressions [35,36]. At the same time, the activation of AMPK and SIRT1 can alleviate the microglial cell-mediated neuroinflammation, as demonstrated by Velagapudi et al. [37]. From our models, a significant decrease was detected in the protein expression of NF-κB p65 in DHM-treated AD rats. In agreement, the memory deficits and the increased inflammatory mediators, such as TNF-α and IL-1β, were observed by Gao et al. in D-gal-induced AD rats, which was closely associated with SIRT1/NF-κB signaling pathway [38], further indicating that DHM may ameliorate the cognitive functions and inhibit the neuroinflammatory responses through activation of AMPK/SIRT1 signaling pathway to down-regulate NF-κB p65.

At the same time, the decreased Bcl-2 (anti-apoptotic protein) and increased Bax (pro-apoptotic protein) were identified in AD rats induced by Aβ_1–42_ but were reversed after treatment with either 100 or 200 mg/kg of DHM. TUNEL staining further confirmed a significant elevation in the hippocampal apoptosis of AD rats, which was rescued after DHM administration, indicating that DHM may alleviate neuronal injuries and hippocampal apoptosis of AD rats. As reported, neuronal apoptosis is a key cause for AD, and down-regulation of Bcl-2 has been thought to be related to tangle-bearing neurons in AD brains [39,40]. Kou et al. [41] also noted that DHM can decrease the p53-mediated cell apoptosis through miR-34a-mediated SIRT1/mTOR signaling pathway, resulting in the down-regulation of Caspase-3 and promotion of Bcl-2. It has been well-recognized that p53 is not only a common target of SIRT1 [42], but also a critical transcript factor, and its activity enhancement mediates the cell apoptosis [43]. In light of this, we supposed that DHM may activate the AMPK/SIRT signaling pathway to improve the neuronal apoptosis possibly through the inhibition of p53-mediated apoptosis signaling pathway.

In conclusion, DHM can activate the AMPK/SIRT1 pathway to inhibit the inflammatory responses and hippocampal neuronal apoptosis, and ameliorates learning deficits of AD rats, which provides novel targets for the clinical treatment of AD.

## Supporting information

**Supplementary Figure 1:** 

## Abbreviations

Aβ: amyloid-β
Aβ_1–42_: amyloid-β_1–42_
AD: Alzheimer’s disease
AMP: ampelopsin
AMPK: AMP-activated protein kinase
APP: amyloid precursor protein
DHM: Dihydromyricetin
IL: interleukin
NF-κB: nuclear factor-κB
PFA: paraformaldehyde
TNF: tumor necrosis factor

## References

[1] Huang T.C., Lu K.T., Wo Y.Y., Wu Y.J. and Yang Y.L. (2011) Resveratrol protects rats from Abeta-induced neurotoxicity by the reduction of iNOS expression and lipid peroxidation. PLoS One 6, e29102 10.1371/journal.pone.0029102 22220203 PMC3248406

[2] Backman L., Jones S., Berger A.K., Laukka E.J. and Small B.J. (2005) Cognitive impairment in preclinical Alzheimer’s disease: a meta-analysis. Neuropsychology 19, 520–531 10.1037/0894-4105.19.4.520 16060827

[3] Taly A., Corringer P.J., Guedin D., Lestage P. and Changeux J.P. (2009) Nicotinic receptors: allosteric transitions and therapeutic targets in the nervous system. Nat. Rev. Drug Discov. 8, 733–750 10.1038/nrd2927 19721446

[4] Beagley K.W., Huston W.M., Hansbro P.M. and Timms P. (2009) Chlamydial infection of immune cells: altered function and implications for disease. Crit. Rev. Immunol. 29, 275–305 10.1615/CritRevImmunol.v29.i4.10 19673684

[5] Wolk D.A. and Klunk W. (2009) Update on amyloid imaging: from healthy aging to Alzheimer’s disease. Curr. Neurol. Neurosci. Rep. 9, 345–352 10.1007/s11910-009-0051-4 19664363 PMC2825106

[6] Ma T., Tan M.S., Yu J.T. and Tan L. (2014) Resveratrol as a therapeutic agent for Alzheimer’s disease. Biomed Res. Int. 2014, 350516 10.1155/2014/350516 25525597 PMC4261550

[7] Escribano L., Simon A.M., Gimeno E., Cuadrado-Tejedor M., Lopez de Maturana R., Garcia-Osta A. (2010) Rosiglitazone rescues memory impairment in Alzheimer’s transgenic mice: mechanisms involving a reduced amyloid and tau pathology. Neuropsychopharmacology 35, 1593–1604 10.1038/npp.2010.32 20336061 PMC3055461

[8] Akiyama H., Arai T., Kondo H., Tanno E., Haga C. and Ikeda K. (2000) Cell mediators of inflammation in the Alzheimer disease brain. Alzheimer Dis. Assoc. Disord. 14 Suppl 1, S47–S53 10.1097/00002093-200000001-00008 10850730

[9] Yan J.J., Kim D.H., Moon Y.S., Jung J.S., Ahn E.M., Baek N.I. (2004) Protection against beta-amyloid peptide-induced memory impairment with long-term administration of extract of Angelica gigas or decursinol in mice. Prog. Neuropsychopharmacol. Biol. Psychiatry 28, 25–30 14687853 10.1016/S0278-5846(03)00168-4

[10] Klyubin I., Cullen W.K., Hu N.W. and Rowan M.J. (2012) Alzheimer’s disease Abeta assemblies mediating rapid disruption of synaptic plasticity and memory. Mol. Brain 5, 25 10.1186/1756-6606-5-25 22805374 PMC3502131

[11] Kou X., Shen K., An Y., Qi S., Dai W.X. and Yin Z. (2012) Ampelopsin inhibits H(2)O(2)-induced apoptosis by ERK and Akt signaling pathways and up-regulation of heme oxygenase-1. Phytother. Res. 26, 988–994 10.1002/ptr.3671 22144097

[12] Shi L., Zhang T., Liang X., Hu Q., Huang J., Zhou Y. (2015) Dihydromyricetin improves skeletal muscle insulin resistance by inducing autophagy via the AMPK signaling pathway. Mol. Cell. Endocrinol. 409, 92–102 10.1016/j.mce.2015.03.009 25797177

[13] Liang X., Zhang T., Shi L., Kang C., Wan J., Zhou Y. (2015) Ampelopsin protects endothelial cells from hyperglycemia-induced oxidative damage by inducing autophagy via the AMPK signaling pathway. Biofactors 41, 463–475 10.1002/biof.1248 26644014

[14] Kou X., Li J., Liu X., Yang X., Fan J. and Chen N. (2017) Ampelopsin attenuates the atrophy of skeletal muscle from d-gal-induced aging rats through activating AMPK/SIRT1/PGC-1alpha signaling cascade. Biomed. Pharmacother. 90, 311–320 10.1016/j.biopha.2017.03.070 28364603

[15] Zhu X., Dahlmans V., Thali R., Preisinger C., Viollet B., Voncken J.W. (2016) AMP-activated protein kinase up-regulates mitogen-activated protein (MAP) kinase-interacting serine/threonine kinase 1a-dependent phosphorylation of eukaryotic translation initiation factor 4E. J. Biol. Chem. 291, 17020–17027 10.1074/jbc.C116.740498 27413184 PMC5016107

[16] Burkewitz K., Zhang Y. and Mair W.B. (2014) AMPK at the nexus of energetics and aging. Cell Metab. 20, 10–25 10.1016/j.cmet.2014.03.002 24726383 PMC4287273

[17] Guarente L. (2006) Sirtuins as potential targets for metabolic syndrome. Nature 444, 868–874 10.1038/nature05486 17167475

[18] Shah S.A., Yoon G.H., Chung S.S., Abid M.N., Kim T.H., Lee H.Y. (2017) Novel osmotin inhibits SREBP2 via the AdipoR1/AMPK/SIRT1 pathway to improve Alzheimer’s disease neuropathological deficits. Mol. Psychiatry 22, 407–416 10.1038/mp.2016.23 27001618 PMC5322276

[19] Bayne K. (1996) Revised Guide for the Care and Use of Laboratory Animals available. American Physiological Society. Physiologist 39, 199, 208-111 8854724

[20] Sheng C., Xu P., Zhou K., Deng D., Zhang C. and Wang Z. (2017) Icariin attenuates synaptic and cognitive deficits in an abeta1-42-induced rat model of Alzheimer’s disease. Biomed. Res. Int. 2017, 7464872 10.1155/2017/7464872 29057264 PMC5625750

[21] Alzoubi K.H., Alhaider I.A., Tran T.T., Mosely A. and Alkadhi K.K. (2011) Impaired neural transmission and synaptic plasticity in superior cervical ganglia from beta-amyloid rat model of Alzheimer’s disease. Curr. Alzheimer Res. 8, 377–384 10.2174/156720511795745311 21453246

[22] Zhang L., Fang Y., Lian Y., Chen Y., Wu T., Zheng Y. (2015) Brain-derived neurotrophic factor ameliorates learning deficits in a rat model of Alzheimer’s disease induced by abeta1-42. PLoS One 10, e0122415 10.1371/journal.pone.0122415 25849905 PMC4388634

[23] Yan J.J., Cho J.Y., Kim H.S., Kim K.L., Jung J.S., Huh S.O. (2001) Protection against beta-amyloid peptide toxicity in vivo with long-term administration of ferulic acid. Br. J. Pharmacol. 133, 89–96 10.1038/sj.bjp.0704047 11325798 PMC1572763

[24] Bi B.T., Lin H.B., Cheng Y.F., Zhou H., Lin T., Zhang M.Z. (2012) Promotion of beta-amyloid production by C-reactive protein and its implications in the early pathogenesis of Alzheimer’s disease. Neurochem. Int. 60, 257–266 10.1016/j.neuint.2011.12.007 22202667

[25] Marlow L., Cain M., Pappolla M.A. and Sambamurti K. (2003) Beta-secretase processing of the Alzheimer’s amyloid protein precursor (APP). J. Mol. Neurosci. 20, 233–239 10.1385/JMN:20:3:233 14501002

[26] Xu P.X., Wang S.W., Yu X.L., Su Y.J., Wang T., Zhou W.W. (2014) Rutin improves spatial memory in Alzheimer’s disease transgenic mice by reducing Abeta oligomer level and attenuating oxidative stress and neuroinflammation. Behav. Brain Res. 264, 173–180 10.1016/j.bbr.2014.02.002 24512768

[27] Dong X., Zhang D., Zhang L., Li W. and Meng X. (2012) Osthole improves synaptic plasticity in the hippocampus and cognitive function of Alzheimer’s disease rats via regulating glutamate. Neural Regen. Res. 7, 2325–2332 25538756 10.3969/j.issn.1673-5374.2012.30.001 PMC4268736

[28] Wang Y., Cella M., Mallinson K., Ulrich J.D., Young K.L., Robinette M.L. (2015) TREM2 lipid sensing sustains the microglial response in an Alzheimer’s disease model. Cell 160, 1061–1071 10.1016/j.cell.2015.01.049 25728668 PMC4477963

[29] Kamphuis W., Kooijman L., Orre M., Stassen O., Pekny M. and Hol E.M. (2015) GFAP and vimentin deficiency alters gene expression in astrocytes and microglia in wild-type mice and changes the transcriptional response of reactive glia in mouse model for Alzheimer’s disease. Glia 63, 1036–1056 10.1002/glia.22800 25731615

[30] Savarin C., Hinton D.R., Valentin-Torres A., Chen Z., Trapp B.D., Bergmann C.C. (2015) Astrocyte response to IFN-gamma limits IL-6-mediated microglia activation and progressive autoimmune encephalomyelitis. J. Neuroinflammation 12, 79 10.1186/s12974-015-0293-9 25896970 PMC4410573

[31] Doens D. and Fernandez P.L. (2014) Microglia receptors and their implications in the response to amyloid beta for Alzheimer’s disease pathogenesis. J. Neuroinflammation 11, 48 10.1186/1742-2094-11-48 24625061 PMC3975152

[32] Weng L., Zhang H., Li X., Zhan H., Chen F., Han L. (2017) Ampelopsin attenuates lipopolysaccharide-induced inflammatory response through the inhibition of the NF-kappaB and JAK2/STAT3 signaling pathways in microglia. Int. Immunopharmacol. 44, 1–8 10.1016/j.intimp.2016.12.018 27998743

[33] Xue B., Yang Z., Wang X. and Shi H. (2012) Omega-3 polyunsaturated fatty acids antagonize macrophage inflammation via activation of AMPK/SIRT1 pathway. PLoS One 7, e45990 10.1371/journal.pone.0045990 23071533 PMC3465287

[34] Ren Z., Cui J., Huo Z., Xue J., Cui H., Luo B. (2012) Cordycepin suppresses TNF-alpha-induced NF-kappaB activation by reducing p65 transcriptional activity, inhibiting IkappaBalpha phosphorylation, and blocking IKKgamma ubiquitination. Int. Immunopharmacol. 14, 698–703 10.1016/j.intimp.2012.10.008 23102662

[35] Baker R.G., Hayden M.S. and Ghosh S. (2011) NF-kappaB, inflammation, and metabolic disease. Cell Metab. 13, 11–22 10.1016/j.cmet.2010.12.008 21195345 PMC3040418

[36] Matsushita T., Sasaki H., Takayama K., Ishida K., Matsumoto T., Kubo S. (2013) The overexpression of SIRT1 inhibited osteoarthritic gene expression changes induced by interleukin-1beta in human chondrocytes. J. Orthop. Res. 31, 531–537 10.1002/jor.22268 23143889

[37] Velagapudi R., El-Bakoush A., Lepiarz I., Ogunrinade F. and Olajide O.A. (2017) AMPK and SIRT1 activation contribute to inhibition of neuroinflammation by thymoquinone in BV2 microglia. Mol. Cell. Biochem. 435, 149–162 10.1007/s11010-017-3064-3 28551846 PMC5632349

[38] Gao J., Zhou R., You X., Luo F., He H., Chang X. (2016) Salidroside suppresses inflammation in a D-galactose-induced rat model of Alzheimer’s disease via SIRT1/NF-kappaB pathway. Metab. Brain Dis. 31, 771–778 10.1007/s11011-016-9813-2 26909502

[39] Xian Y.F., Mao Q.Q., Wu J.C., Su Z.R., Chen J.N., Lai X.P. (2014) Isorhynchophylline treatment improves the amyloid-beta-induced cognitive impairment in rats via inhibition of neuronal apoptosis and tau protein hyperphosphorylation. J. Alzheimers Dis. 39, 331–346 10.3233/JAD-131457 24164737

[40] Su J.H., Deng G. and Cotman C.W. (1997) Bax protein expression is increased in Alzheimer’s brain: correlations with DNA damage, Bcl-2 expression, and brain pathology. J. Neuropathol. Exp. Neurol. 56, 86–93 10.1097/00005072-199701000-00009 8990132

[41] Kou X., Liu X., Chen X., Li J., Yang X., Fan J. (2016) Ampelopsin attenuates brain aging of D-gal-induced rats through miR-34a-mediated SIRT1/mTOR signal pathway. Oncotarget 7, 74484–74495 10.18632/oncotarget.12811 27780933 PMC5342681

[42] Xiong H., Pang J., Yang H., Dai M., Liu Y., Ou Y. (2015) Activation of miR-34a/SIRT1/p53 signaling contributes to cochlear hair cell apoptosis: implications for age-related hearing loss. Neurobiol. Aging 36, 1692–1701 10.1016/j.neurobiolaging.2014.12.034 25638533

[43] Haupt S., Berger M., Goldberg Z. and Haupt Y. (2003) Apoptosis - the p53 network. J. Cell Sci. 116, 4077–4085 10.1242/jcs.00739 12972501

