# Supplementary figures and images for: Protective role of Dihydromyricetin in Alzheimer’s disease rat model associated with activating AMPK/SIRT1 signaling pathway
Source: Biosci Rep. 2019 Jan 3;39(1):BSR20180902. doi: 10.1042/BSR20180902 (PMC6328867; doi:10.1042/BSR20180902)

Volts

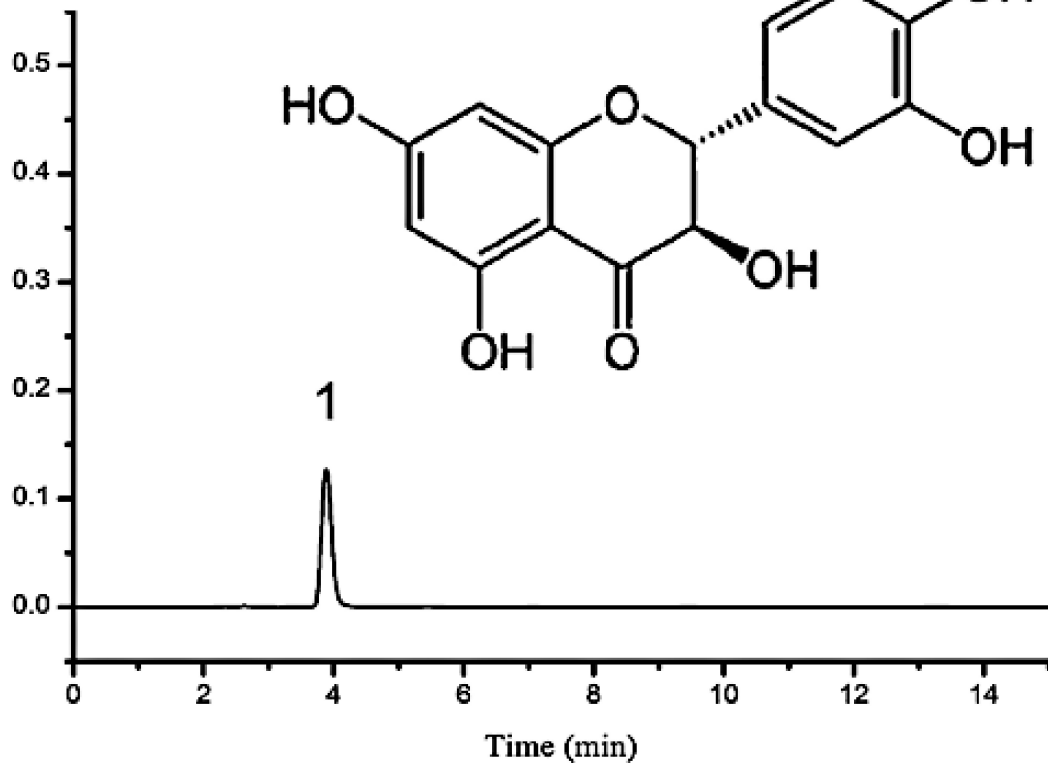

Supplement: Supplementary file 1 [file bsr20180902_Supp1.pdf]
